# Harvard Odontological Society

**Published:** 1891-07

**Authors:** 

**Affiliations:** Harvard Odontological Society


### HARVARD ODONTOLOGICAL SOCIETY.

The Harvard Odontological Society held its regular monthly meeting December 31, 1890, at Young's Hotel, Boston. President Cooke in the chair.

The paper for the evening was by J. G. W. Werner, D.M.D. Subject, "A Case in Practice."[1]

(For Dr. Werner's paper, see page 105.)

*Dr. Hitchcock.*—Dr. Werner has given such a full account of this case that there is little for me to add. In the paper which he read to-night he spoke of the lady having a chill on the third day. Had I known this at the time, I should have refused to take it, knowing it indicated septicæmia. I think it was the second time I saw her that she spoke of having the chill. Gave her quinine, telling her that if another occurred to commence using it at once.

The chief causes of these imprisoned teeth are the want of the necessary space for proper eruption, retarded evolution, striking against teeth, and retarded absorption.

We find the first trace of the third molar at twelve years of age, so it is six years in forming and getting into line, and this at the age when there is a special drain on the system. Magitot says, "There is perhaps no organ which yields to the influence of the general condition of the constitution and the change of health more easily than the teeth." The third molar is the tooth which is most likely to be imprisoned,—the inferior oftener than the superior. Next in frequency are the cuspids. These are sometimes revealed by their being partially uncovered through a process of alveolar absorption late in life. As the fourth molar is almost entirely extinct in this generation, so the third molar will undoubtedly be generations hence, as even now the arch is much contracted. Teeth far away from the alveolar arch are quite rare, and when found, are generally encysted. According to Dr. Bolles, the third molars most frequently exhibit this anomaly; next in frequency are the cuspids. Sayre relates a case of a little girl under his care who had three molars separately ejected through the meatus auditorius externus. That the third molar causes a vast amount of trouble is known to us all, simply its eruption often causing serious illness. Esquinol reports the case of a woman who recovered from insanity,

[1] The paper of Dr. Werner should have been followed in the same number by this discussion; but the latter was not received until June. The subject of the paper was a "Case of Impaction of the Third Molar."—[Ed.]

after a crucial incision was made in the gum to promote the eruption of the third molar. Also, three cases of mania in young girls, which only disappeared after the eruption of retarded teeth. M. Vellerix records the case of a young girl of strong constitution and previous good health who had so much irritation and inflammation from the simultaneous eruption of third molars at the angle of the jaw that nausea and irritability of the stomach set in, followed by vomiting, strabismus, convulsions, and death. Lancing had no effect. Dr. Salter relates a case of paralysis of the arm caused by the third molar. Uterine affections, retarded labor, impeded healing of wounds and ulcers, headaches, hip-diseases, and many other disturbances are cited as caused by the teeth.

The question arises, How far are we as dentists to proceed in such operations? Many of those done by the surgeon could have been accomplished with far less cutting, and really making a minor operation out of what is sometimes considered by the surgeons to be a very complicated one. I saw a case a few weeks ago where a bony tumor, one and one-half inches long by one wide, was removed from the superior maxilla by a dentist, he simply making an incision in the gum and the soft parts along the alveolar ridge, removing the tumor from the inside. The patient was sent to the hospital where the surgeon was to cut, beginning on the outside of the face, then go through the jaw, and take out a good-sized piece of that in order to remove the tumor. The operation by the dentist was a minor one, a very serious one if performed by the surgeon, and as a result, of great importance, the leaving of no visible scar.

I had a case in my own practice of necrosis of the palatal bone, it being involved to the extent of about the size of a five-cent piece. On making a thorough examination, I found there was necrosis, and with the assistance of a surgeon (whom I supposed would perform the operation) she was etherized. I had brought my dental engine, thinking it might be of service. After the patient was etherized the surgeon told me to proceed. This surprised me, as I had not the remotest idea that I was to do anything more than to assist. The operation required about half an hour, a fair-sized portion of the palatal bone being removed. The wound was treated regularly and healed very nicely. About three years afterwards, I found the trouble was recurring again slightly. My impression, when first operating upon the case, was that it was caused by an ill-fitting plate which the lady wore, and a new plate was made which was free from friction, and was therefore puzzled on seeing the trouble reappear. In a short time a cuspid tooth which had been imprisoned erupted. With the removal of this tooth the lesion entirely disappeared.

The dentist has the advantage of the surgeon in that he has, or should have, a nicety of touch that will tell him what he is in contact with without seeing it, and whether it is entirely removed. In cases of necrosis a small opening may be made, and the engine-bur will remove the diseased bone far better than scraper and chisel. But suppose we sever the nerve, and paralysis follows? If in a surgeon's hand, probably little blame would be attached; it would count as a sequence of the operation; but in our hands, the operation being considered a minor one, we are judged accordingly. How far should we go?

*Dr. Clapp.*—This is a very interesting case,—we are all liable to get such. I do not quite understand the description of the treatment, and would like to ask Dr. Werner if he considered, when he removed this tooth, that he had removed all the cause of the difficulty at that time?

*Dr. Werner.*—We did not think we had finished the operation by simply removing the tooth, though we considered that to be the cause of the lesion, but after exploring with probe, scraping roughened surfaces of bone, and thoroughly cleansing the cavity, we expected the case to get well, provided the orifice were kept open, the cavity syringed and kept in an aseptic condition, allowing it to heal only from the bottom.

*Dr. Clapp.*—With me the question would have been in regard to the after-treatment, the advisability of keeping the opening packed with cotton. It seems to me there was no question but that the cause of the difficulty was this impacted tooth, and that the presence of the cotton, under these circumstances, would be rather a detriment to the healing up of the wound. If it had not been packed, the opening would not have closed for some little time, and a simple drainage-tube could have been put in without causing any irritation, and there would have been less liability to septicæmia. I do not wish to be understood that I desire to criticise the treatment of the case, but if the case had been in my hands, I should have left the cotton out.

Another thing occurs to me. From his paper I understand that the gentleman thought he was removing a supernumerary tooth. In my opinion this is not a supernumerary tooth, but a retarded third molar.

*Dr. Hitchcock.*—As far as the drainage-tube was concerned, the cotton was not packed so that the orifice was completely closed. It was simply dipped in this solution and placed there loosely, keeping it sweet and clean. Being treated in this way, the wound had all the advantages of capillary attraction that it would have if a drainage-tube had been inserted. If the cotton had been packed in tightly, of course it would not have allowed the wound to heal.

*Dr. Clapp.*—The difference would have been the irritating qualities of the cotton. I have had several cases of large cavities caused by breaking down of the arch, with formation of pus, both in the superior and inferior maxillæ, and I have been afraid of overtreating, fearing they would, if I treated them every day or every other day, sufficiently to cause a fresh wound over the whole surface, be very much slower in healing. By too frequent treatment the granulation that is going on is broken up and the healing is much retarded. That is the theory that I have acted upon. I have found that by infrequent treatment, but keeping the orifice open, much better results are attained.

Impacted teeth—teeth that have failed to present themselves at the proper time—are not very uncommon. They are often there without causing much trouble. Many years ago, when I first began practice here in Boston,—it was probably in 1871 or 1872,—I extracted a badly-decayed superior right lateral for an elderly lady, and I supposed from the appearance that I had broken the tooth. I afterwards took, or tried to take, out what I supposed to be the remainder of the tooth. After considerable trouble, I succeeded in extracting as handsome a cuspid tooth as I have ever seen.

This month I had occasion to examine a patient's mouth, a gentleman who is probably between fifty-five and sixty, who has been wearing a lower denture. By chance I discovered in the right inferior jaw, near the distal portion, a little point that appeared to cover a root of a tooth. The gums in the lower part of the mouth seemed to be almost entirely healthy, and it was by accident that I noticed this little point, and I took up a probe to see if there was a root there. You can imagine my astonishment when I found what was no doubt a wisdom tooth. It was giving him no trouble, and I did not propose extraction to him. It is my opinion that the left inferior wisdom tooth is there also.

*Dr. Briggs.*—I would like to ask Dr. Werner what he considers this large fragment to be?

*Dr. Werner.*—I am in doubt. It is difficult to tell whether it is a portion of the maxilla, the sphenoid, or the palate bone.

*Dr. Briggs.*—It looks to me very much like the two roots of a bicuspid.

*Dr. Smith.*—That is what suggested itself to me.

*President Cooke.*—You could tell by having a section made of it.

*Dr. Werner.*—It is so altered and distorted by disease as to make it impossible for the Professor of Anatomy at Harvard Medical School (to whom I took it) to say what it is. I do not believe it is a root or two roots, and have never for a moment thought it was. The location from which it came was posterior in too great a degree to suggest that it might be tooth substance.

*Dr. Stoddard.*—I saw a case last summer of an imprisoned tooth which was causing a good deal of discomfort. It was a case of Dr. Allan's. The patient was a lady of middle age, who had been wearing an upper plate, all of the upper teeth having been extracted. An irritation was set up and there had been some slight discharge of pus from a small orifice, about half an inch long, back of the border of the alveolus and near the median line. By probing we could feel a little point which we thought was the enamel of a tooth, and after making a longitudinal incision the tooth was taken out and found to be a cuspid, which was lying along the median line with the crown pointing towards the alveolus, about half an inch from the front.

*Dr. Briggs.*—Regarding that matter of the treatment, I think we all understand the object of packing with cotton. It is so easy for the mucous membrane to heal over that it is very important that the orifice should be kept open as long as there is a cavity there. If the opening is allowed to heal too quickly it may enclose in the cavity a few drops of pus, and then the pus will continue to burrow, thereby increasing the diseased portion. That is one of the cases where I have found the antiseptic sponge of great value. It acts to keep the parts open without causing any irritation, and it also serves as a very good drainage-tube. I have usually inserted a long piece at first and shorten it daily as the wound progresses, and I have found it to be an excellent thing to keep the fistula open and allow it to heal from the bottom.

*Dr. Werner.*—In this particular case a drainage-tube was out of the question. It could not have been retained,—it would have fallen into the throat. The shape of the cavity was such that it required a peculiarly constructed tube. I think the antiseptic sponge would have done well, but I did not have it on hand at the time, and cotton served the purpose. It was dipped in oil of cloves and listerine, was tied in the centre with floss silk, the end being allowed to hang down, and was more comfortable than a tube could have been. The patient was most conscientious and faithful in the treatment, keeping the wound clean as a surgeon could have done, packing it carefully several times a day according to the instructions given her. This packing was by degrees diminished until the orifice was allowed to heal up. The tissues healed phenomenally fast for an old person,—she had a good constitution, had never been sick, and was in the best of health.

I think this is one of those cases where the dentist did a conservative operation without the sacrifice of great quantities of bone tissue. It is my belief that if a general surgeon had operated, he would have obtained a good result, but by more heroic treatment, I think, the jaw would have been removed.

*Dr. Clifford.*—I would like to inquire of Dr. Werner what kind of probe he used. I find difficulty when treating such cases in getting a suitable one at the dental depots.

*Dr. Werner.*—I find two or three probes very essential, but in this case I first discovered the orifice with a fine burnisher, which had a handle tapering with a curve not a right angle. It made the best probe I could have procured for the purpose.

*Dr. Hitchcock.*—Most of the probes at the surgical instrument-stores are too large. I have found that a fine silver wire answers the purpose admirably.

H. L. Upham, D.M.D.,
*Editor Harvard Odontological Society.*

